# Energy‐based automatic determination of buffer region in the divide‐and‐conquer second‐order Møller–Plesset perturbation theory

**DOI:** 10.1002/jcc.26486

**Published:** 2021-02-03

**Authors:** Toshikazu Fujimori, Masato Kobayashi, Tetsuya Taketsugu

**Affiliations:** ^1^ Graduate School of Chemical Sciences and Engineering Hokkaido University Sapporo Japan; ^2^ Department of Chemistry, Faculty of Science Hokkaido University Sapporo Japan; ^3^ WPI‐ICReDD Hokkaido University Sapporo Japan; ^4^ ESICB, Kyoto University Kyoto Japan

**Keywords:** divide‐and‐conquer method, electron correlation, fragmentation, Laplace transformed MP2, linear‐scaling calculation

## Abstract

In the linear‐scaling divide‐and‐conquer (DC) electronic structure method, each subsystem is calculated together with the neighboring buffer region, the size of which affects the energy error introduced by the fragmentation in the DC method. The DC self‐consistent field calculation utilizes a scheme to automatically determine the appropriate buffer region that is as compact as possible for reducing the computational time while maintaining acceptable accuracy (*J. Comput. Chem*. **2018**, *39*, 909). To extend the automatic determination scheme of the buffer region to the DC second‐order Møller–Plesset perturbation (MP2) calculation, a scheme for estimating the subsystem MP2 correlation energy contribution from each atom in the buffer region is proposed. The estimation is based on the atomic orbital Laplace MP2 formalism. Based on this, an automatic buffer determination scheme for the DC‐MP2 calculation is constructed and its performance for several types of systems is assessed.

## Graphical abstract

In the linear‐scaling divide‐and‐conquer (DC) electronic structure method, each subsystem is calculated together with the neighboring buffer region, the size of which affects the energy error introduced by the fragmentation. The authors have proposed a scheme to automatically determine the appropriate buffer region for DC Hartree–Fock (HF) calculation that is as compact as possible for reducing the computational time while maintaining acceptable accuracy. They extend the automatic buffer determination scheme to DC second‐order Møller–Plesset perturbation (MP2) calculation. In this scheme, the buffer atoms for DC‐MP2 calculation are selected from those for DC‐HF based on the estimated correlation energy contribution.

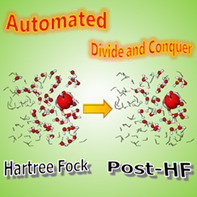

## 1 INTRODUCTION

By virtue of recent advances in quantum chemical theory as well as the improvements in computer performance, electronic structure calculations of large‐scale systems such as proteins have now become technically feasible. Such theoretical advances include the development of linear‐scaling (or low‐scaling) electronic structure methods. In the standard formalism of electronic structure methods, the computational time increases cubically [*O*(*N* ^3^)] with respect to the system size *N*, even with the simplest Hartree–Fock (HF) method^1^ or density functional theory (DFT),^2^ owing to the diagonalization of the Hamiltonian matrix. Furthermore, in case of post‐HF calculations, such as the second order Møller–Plesset perturbation (MP2)^3^, ^4^, ^5^ and coupled cluster (CC) theories,^4^, ^5^ their time scalings deteriorate as *O*(*N* ^5^) or more. Therefore, the standard formalisms of electronic structure methods cannot be applied to large‐scale systems. By introducing approximations to the standard formalisms, many low‐scaling electronic structure methods^6^, ^7^, ^8^, ^9^, ^10^ have been proposed for treating such systems. Many of these methods equip some schemes to adjust the errors derived from the low‐scaling approximations based on the distance parameter. For example, in the molecular tailoring approach proposed by Garde et al.,^11^ *R*‐goodness parameter is used to determine the quality of each fragment.^12^, ^13^ In the generalized energy‐based fragmentation approach,^14^, ^15^ each fragment is constructed with the distance threshold (*ξ*). The cluster‐in‐molecule local correlation method also adopts the distance threshold *ξ* to control the size of the cluster,^16^ while a simple correction scheme to account for the distant‐pair correlation has recently been proposed.^17^ The accuracy of the fragment molecular orbital method^18^ can be systematically improved by increasing the order of many‐body expansion from the original two‐body to three‐body^19^, ^20^ and four‐body^21^ expansions. The pair natural orbital (PNO) electron correlation approach^22^, ^23^ adopts several truncation schemes for construction of correlated virtual orbitals (i.e., PNOs) for each occupied local molecular orbital (MO) pair, where the bond‐based (so‐called IEXT) or distance‐based (so‐called REXT) truncation is used to determine the local virtual orbital region to construct PNOs. Since molecular energy is the most important property in quantum chemical calculations, an energy‐based parameter is more desirable than a distance‐based one. For example, the divide‐expand‐consolidate method utilizes the energy‐based fragment optimization threshold to determine the atomic occupied and virtual orbital spaces in each fragment.^24^, ^25^

Yang and coworkers introduced a linear‐scaling approach called the divide‐and‐conquer (DC) method.^26^, ^27^ The DC method has been applied to the HF or DFT self‐consistent field (SCF),^26^, ^28^ density‐functional tight‐binding,^29^, ^30^, ^31^, ^32^ and post‐HF (MP2^33^, ^34^, ^35^, ^36^ or CC^37^, ^38^, ^39^) energy calculations as well as the SCF^40^ and MP2^41^ energy gradient calculations. For treating static electron correlation in large‐scale systems, the DC method has also been combined with the Hartree–Fock–Bogoliubov method^42^ and the thermally‐assisted occupation (finite temperature) scheme.^43^ In the DC method, the size of the buffer region plays the role of the distance parameter to adjust the approximation error; a larger buffer size leads to a smaller approximation error. However, it is still difficult to estimate the error in energy based on the distance‐based adjustment parameter. Recently, we^44^ proposed a scheme to estimate the energy error introduced in the DC‐HF and DC‐DFT calculations using a two‐layer buffer region scheme introduced by Dixon and Merz.^45^ This estimation scheme can successfully be applied to automatically determine the appropriate buffer region based on the estimated energy error.^44^

This study attempts to export the idea of the previous automated DC‐HF scheme to the DC‐MP2 calculation. Kobayashi et al.^36^ reported that the buffer region used for the MP2 correlation calculation can be contracted from that for the HF one to achieve the same energy accuracy as the DC‐HF calculation because of the short‐range nature of the MP2 dynamical electron correlation. We first develop a method to estimate the subsystem MP2 correlation energy contribution from each atom in the buffer region. Here, the idea of the atomic orbital (AO) Laplace MP2 method^46^, ^47^, ^48^, ^49^, ^50^ is used as well as the Schwarz inequality. Based on this estimated energy contribution, we established an algorithm to automatically determine the appropriate buffer region in the DC‐MP2 calculation.

This paper consists of four sections. Section 2 gives a brief summary of the linear‐scaling DC electron correlation method with a fixed buffer region as well as the present procedure to estimate the energy contribution from each buffer atom and the automated DC‐MP2 algorithm. Numerical assessments are described in Section 3. Finally, we provide concluding remarks in Section 4.

## 2 METHODS

### 2.1 The DC‐MP2 electron correlation calculation

We first outline the DC‐MP2 electron correlation calculation scheme. The DC‐MP2 method is applicable only with atom‐centered basis functions. Each basis function, *ϕ*_*μ*_(**r**), called an AO, is denoted by a Greek letter index, *μ*, *ν*, …. In the DC method, the entire system is divided into several subsystems, each of which consists of the central and buffer regions. Each central region is mutually exclusive with the other central regions. The sets of AOs belonging to the central and buffer regions of subsystem *α* are referred to as ***S***(*α*) and ***B***(*α*), respectively.

In the DC‐MP2 method, the MOs in the subsystem *α*,

(1) $$\psi_{p}^{\alpha }\left(r\right)=\sum_{\mu \;\in \;L\left(\alpha \right)}C_{\mathrm{\mu p}}^{\alpha }\phi_{\mu }\left(r\right)$$

are used to evaluate the correlation energy of subsystem *α*, where ***L***(*α*) = ***S***(*α*) ∪ ***B***(*α*) represents the set of AOs in the localization region and *p* refers to an arbitrary MO. The MO coefficients, $\left\{C_{p}^{\alpha }\right\}$, and the MO energies, $\left\{\varepsilon_{p}^{\alpha }\right\}$, of subsystem *α* are obtained by solving the Roothaan equation for each subsystem:

(2) $$F^{\alpha }\left[D^{\mathrm{SCF}}\right]C_{p}^{\alpha }=\varepsilon_{p}^{\alpha }S^{\alpha }C_{p}^{\alpha }$$

where **F** ^*α*^[**D** ^SCF^] is the subsystem Fock matrix constructed with the density matrix **D** ^SCF^, and **S** ^*α*^ is the subsystem overlap matrix with the element $S_{\mu \nu }^{\alpha }=\left(\phi_{\mu }|\phi_{\nu }\right)$ for *μ*, *ν* ∈ ***L***(*α*). Note that the subsystem Roothaan equation (_2_) has to be solved not self‐consistently but just once using predetermined **D** ^SCF^. The density matrix, **D** ^SCF^, can be constructed from the standard or approximate HF calculation, such as the DC‐HF one. If **D** ^SCF^ is obtained from the DC‐HF calculation, it is constructed with the local density matrices, {**D**^*α*^}, and the partition matrices, {**p**^*α*^}, as the following:

(3) $$D_{\mu \nu }^{\mathrm{SCF}}\approx \sum_{\alpha }^{\text{subsystem}}p_{\mu \nu }^{\alpha }D_{\mu \nu }^{\alpha }$$

(4) $$D_{\mu \nu }^{\alpha }=\sum_{p}f_{\beta }\left(\varepsilon_{F}-\varepsilon_{p}^{\alpha }\right)C_{\mathrm{\mu p}}^{\alpha }C_{\mathrm{\nu p}}^{\alpha }$$

(5) $$p_{\mu \nu }^{\alpha }=\left\{\begin{array}{cc} 1 & \left(\mu \;\in \;S\left(\alpha \right)\wedge \nu \;\in \;S\left(\alpha \right)\right)\\ 1/2 & \left(\left(\mu \;\in \;S\left(\alpha \right)\wedge \nu \;\in \;B_{\mathrm{SCF}}\left(\alpha \right)\right)\vee \left(\mu \;\in \;B_{\mathrm{SCF}}\left(\alpha \right)\wedge \nu \;\in \;S\left(\alpha \right)\right)\right)\\ 0 & \left(\text{otherwise}\right) \end{array}\right)$$

where *f*_*β*_(*x*) = [1 + exp(−*βx*)]^−1^ is the Fermi distribution function with the inverse temperature, *β*, and *ε*_F_ is the universal Fermi level. The details of the DC‐HF procedure can be found in Reference 27, for example.

Before the evaluation of the subsystem correlation energy, the subsystem MOs of Equation (1) must be classified into occupied $\left\{\psi_{i}^{\alpha },\psi_{j}^{\alpha },\ldots \right\}$ and virtual ones $\left\{\psi_{a}^{\alpha },\psi_{b}^{\alpha },\ldots \right\}$. This can be accomplished by, for example, using the Fermi level determined in the prior DC‐HF calculations. The MP2 correlation energy for the entire system, $\Delta E_{\text{corr}}^{\left(2\right)}$, can be approximated as the sum of the subsystem MP2 correlation energies, $\left\{\Delta E_{\text{corr}}^{\alpha \left(2\right)}\right\}$,

(6) $$\Delta E_{\text{corr}}^{\left(2\right)}\approx \sum_{\alpha }\Delta E_{\text{corr}}^{\alpha \left(2\right)}$$

Because the buffer region in each localization region overlaps with the other localization regions, $\Delta E_{\text{corr}}^{\alpha \left(2\right)}$ is obtained as the MP2 correlation energy corresponding to the central region of the localization region *α* by means of energy density analysis (EDA).^51^ The subsystem correlation energy is then evaluated by

(7) $$\Delta E_{\text{corr}}^{\alpha \left(2\right)}=\sum_{i^{\alpha },j^{\alpha }}^{\mathrm{occ}\left(\alpha \right)}\sum_{a^{\alpha },b^{\alpha }}^{\mathrm{vir}\left(\alpha \right)}\sum_{\mu \;\in \;S\left(\alpha \right)}\frac{C_{\mathrm{\mu i}}^{\alpha }\left(\mu a^{\alpha }|j^{\alpha }b^{\alpha }\right)}{\varepsilon_{i}^{\alpha }+\varepsilon_{j}^{\alpha }-\varepsilon_{a}^{\alpha }-\varepsilon_{b}^{\alpha }}\left[2\left(a^{\alpha }i^{\alpha }|b^{\alpha }j^{\alpha }\right)-\left(a^{\alpha }j^{\alpha }|b^{\alpha }i^{\alpha }\right)\right]$$

with the two‐electron integral notation

$$\left(i^{\alpha }a^{\alpha }|j^{\alpha }b^{\alpha }\right)=\iint dr_{1}dr_{2}\psi_{i}^{\alpha *}\left(r_{1}\right)\psi_{a}^{\alpha }\left(r_{1}\right)r_{12}^{-1}\psi_{j}^{\alpha *}\left(r_{2}\right)\psi_{b}^{\alpha }\left(r_{2}\right)$$

### 2.2 Estimation of the DC‐MP2 energy

Based on EDA, the MP2 correlation energy for subsystem *α*, $\Delta E_{\text{corr}}^{\alpha \left(2\right)}$, can be further divided into contributions from the atoms in the localization region *α*, $\Delta E_{B}^{\alpha \left(2\right)}$, as

(8) $$\Delta E_{\text{corr}}^{\alpha \left(2\right)}=\sum_{B\;\in \;L\left(\alpha \right)}\Delta E_{B}^{\alpha \left(2\right)}$$

(9) $$\Delta E_{B}^{\alpha \left(2\right)}=\sum_{i^{\alpha },j^{\alpha }}^{\mathrm{occ}\left(\alpha \right)}\sum_{a^{\alpha },b^{\alpha }}^{\mathrm{vir}\left(\alpha \right)}\sum_{\mu \;\in \;S\left(\alpha \right)}\sum_{\nu \;\in \;B}\frac{C_{\mathrm{\mu i}}^{\alpha }C_{\mathrm{\nu a}}^{\alpha }\left(\mu \nu |j^{\alpha }b^{\alpha }\right)}{\varepsilon_{i}^{\alpha }+\varepsilon_{j}^{\alpha }-\varepsilon_{a}^{\alpha }-\varepsilon_{b}^{\alpha }}\left[2\left(a^{\alpha }i^{\alpha }|b^{\alpha }j^{\alpha }\right)-\left(a^{\alpha }j^{\alpha }|b^{\alpha }i^{\alpha }\right)\right]$$

According to the local correlation philosophy for dynamical electron correlation,^52^, ^53^, ^54^ it is expected that $\Delta E_{B}^{\alpha \left(2\right)}$ rapidly decreases as the distance between atom *B* and central region *α* increases. The exponential decay of the MP2 energy contribution with respect to the interatomic distance is discussed in the Appendix. As pointed out by Kobayashi and Nakai,^36^ the appropriate size of the buffer region for the DC‐MP2 calculation can be smaller than that for the DC‐HF calculation because of the locality of the dynamical electron correlation. Therefore, if the absolute value of $\Delta E_{B}^{\alpha \left(2\right)}$ is estimated to be smaller than some criterion, the energy change by excluding atom *B* from the buffer region of subsystem *α* is expected to be small. By applying the AO‐Laplace MP2 technique to Equation (_9_), $\Delta E_{B}^{\alpha \left(2\right)}$ can be expressed as.

(10) $$\text{Δ}E_{B}^{\alpha \left(2\right)}=-\int_{0}^{\infty }\underset{\mu \;\in \;S\left(\alpha \right)}{\sum }\underset{\nu \;\in \;B}{\sum }\underset{\lambda \sigma }{\sum }\underset{\gamma \kappa \delta \varepsilon }{\sum }X_{\mu \gamma }^{\alpha }\left(\tau \right)Y_{\nu \kappa }^{\alpha }\left(\tau \right)X_{\lambda \delta }^{\alpha }\left(\tau \right)Y_{\sigma \varepsilon }^{\alpha }\left(\tau \right)\left(\mu \nu \text{|}\lambda \sigma \right)\left[2\left(\kappa \gamma \text{|}\varepsilon \delta \right)\text{​}-\left(\kappa \delta \text{|}\varepsilon \gamma \right)\right]d\tau$$

where **X** ^*α*^(*τ*) and **Y** ^*α*^(*τ*) are the energy‐weighted density matrices expressed as

(11) $$X_{\mu \nu }^{\alpha }\left(\tau \right)=\sum_{i}C_{\mathrm{\mu i}}^{\alpha }C_{\mathrm{\nu i}}^{\alpha }e^{\left(\varepsilon_{i}^{\alpha }-\varepsilon_{F}\right)\tau }\;$$

(12) $$Y_{\mu \nu }^{\alpha }\left(\tau \right)=\sum_{a}C_{\mathrm{\mu a}}^{\alpha }C_{\mathrm{\nu a}}^{\alpha }e^{-\left(\varepsilon_{a}^{\alpha }-\varepsilon_{F}\right)\tau }$$

Here, the Fermi level, *ε*_F_, may be already determined in the prior DC‐HF calculation, or may be the midpoint energy between HOMO and LUMO in the prior HF calculation. For estimation purpose, we drastically approximate the integral in Equation (_10_) by the one‐point Gauss–Laguerre quadrature, namely,

(13) $$\Delta E_{B}^{\alpha \left(2\right)}∼-e\sum_{\mu \;\in \;S\left(\alpha \right)}\sum_{\nu \;\in \;B}\sum_{\lambda \sigma }\sum_{\text{γκδε}}X_{\mu \gamma }^{\alpha }Y_{\nu \kappa }^{\alpha }X_{\lambda \delta }^{\alpha }Y_{\sigma \varepsilon }^{\alpha }\left(\mu \nu |\lambda \sigma \right)\left[2\left(\kappa \gamma |\varepsilon \delta \right)-\left(\kappa \delta |\varepsilon \gamma \right)\right]$$

(14) $$X_{\mu \nu }^{\alpha }=\sum_{i}C_{\mathrm{\mu i}}^{\alpha }C_{\mathrm{\nu i}}^{\alpha }e^{\left(\varepsilon_{i}^{\alpha }-\varepsilon_{F}\right)}\;$$

(15) $$Y_{\mu \nu }^{\alpha }=\sum_{a}C_{\mathrm{\mu a}}^{\alpha }C_{\mathrm{\nu a}}^{\alpha }e^{-\left(\varepsilon_{a}^{\alpha }-\varepsilon_{F}\right)}$$

Assuming that the rhs of Equation (_13_) gives the upper limit of $\Delta E_{B}^{\alpha \left(2\right)}$, its absolute value can be bounded by adopting the Schwarz inequality

(16) $$\left|\left(\mathrm{ij}|\mathrm{kl}\right)\right|\leq \sqrt{\left|\left(\mathrm{ij}|\mathrm{ij}\right)\right|}\sqrt{\left|\left(\mathrm{kl}|\mathrm{kl}\right)\right|}$$

as(17)
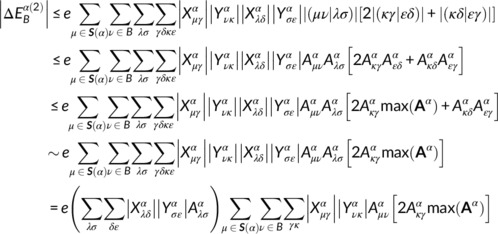
where $A_{\mu \nu }^{\alpha }=\sqrt{\left|\left(\mu \nu |\mu \nu \right)\right|}$. Here, on the analogy to the scaled opposite‐spin MP2 method,^55^ the term $A_{\kappa \delta }^{\alpha }A_{\varepsilon \gamma }^{\alpha }$ was omitted owing to its smaller contribution. Because the summation in parentheses in Equation (_17_) is constant for subsystem *α*, the following index can be considered as the magnitude of the contribution from atom *B*:

(18) $$e_{B}^{\alpha }=e\sum_{\mu \;\in \;S\left(\alpha \right)}\sum_{\nu \;\in \;B}\sum_{\gamma \kappa }\left|X_{\mu \gamma }^{\alpha }\right|\left|Y_{\nu \kappa }^{\alpha }\right|A_{\mu \nu }^{\alpha }\left[2A_{\kappa \gamma }^{\alpha }\max\left(A^{\alpha }\right)\right]$$

Using the above $e_{B}^{\alpha }$ index, we propose the following automatic determination scheme for the buffer region in the DC‐MP2 method:

1. Assignment of the initial DC‐MP2 buffer region for each subsystem. This may be determined by prior DC‐HF calculation.
2. Evaluation of $e_{B}^{\alpha }$ from Equation (_18_).
3. The exclusion of atom *B* from the buffer region of subsystem *α* if $e_{B}^{\alpha }$ is smaller than the energy threshold.
4. Reconstruction of subsystem molecular orbitals $\left\{C_{p}^{\alpha }\right\}$ and $\left\{\varepsilon_{p}^{\alpha }\right\}$, using Equation (_2_).
5. Evaluation of the subsystem correlation energy, $\Delta E_{\text{corr}}^{\alpha \left(2\right)}$, from Equation (_7_).

The additional computational cost for the evaluation of all necessary $e_{B}^{\alpha }$ scales as *O*(*Nm* ^3^), where *N* and *m* represent the sizes of the entire system and buffer region, respectively, since the evaluation of each $e_{B}^{\alpha }$ of Equation (_18_) scales with *O*(*m* ^2^) owing to the summation over *γ* and *κ* and the number of $e_{B}^{\alpha }$ to be evaluated scales with *O*(*Nm*).

## 3 NUMERICAL ASSESSMENTS

### 3.1 Computational details

We implemented the above‐mentioned automatically controlled DC‐MP2 method to the GAMESS package^56^, ^57^ and evaluated its accuracy and efficiency for the different types of systems. In the DC‐HF calculations, the inverse temperature parameter, *β*, was set to 125 a.u. and the Fermi function cutoff factor (the FTOL option of $DANDC input group in GAMESS program) was set to 20. In addition, the parameters in the automated DC‐HF method were set to $e_{\text{thresh}}^{\mathrm{SCF}}$ = 0.1 μ*E*_h_ and *r*_ext_ = 3.0 Å, the definitions of which are given in our previous paper.^44^ The 6‐31G(d) basis set^58^ was adopted throughout this paper. We introduced the major axis radii of the HF and MP2 localization regions for subsystem *α*, $l_{\text{local}}^{\text{SCF},\alpha }$ and $l_{\text{local}}^{\text{corr},\alpha }$, respectively, to discuss the size of the localization regions determined by the automated DC method. $l_{\text{local}}^{\mathrm{SCF},\alpha }$ (or $l_{\text{local}}^{\text{corr},\alpha }$) was defined as half of the maximum atom‐pair distance in the HF (or MP2) localization region for subsystem *α*. The two‐electron AO integrals, (*μν*|*λσ*), were treated in so‐called “direct algorithm” manner, that is, the same integrals were calculated repeatedly for every subsystem.

### 3.2 Estimation of the atomic MP2 energy contributions

**FIGURE 1:**
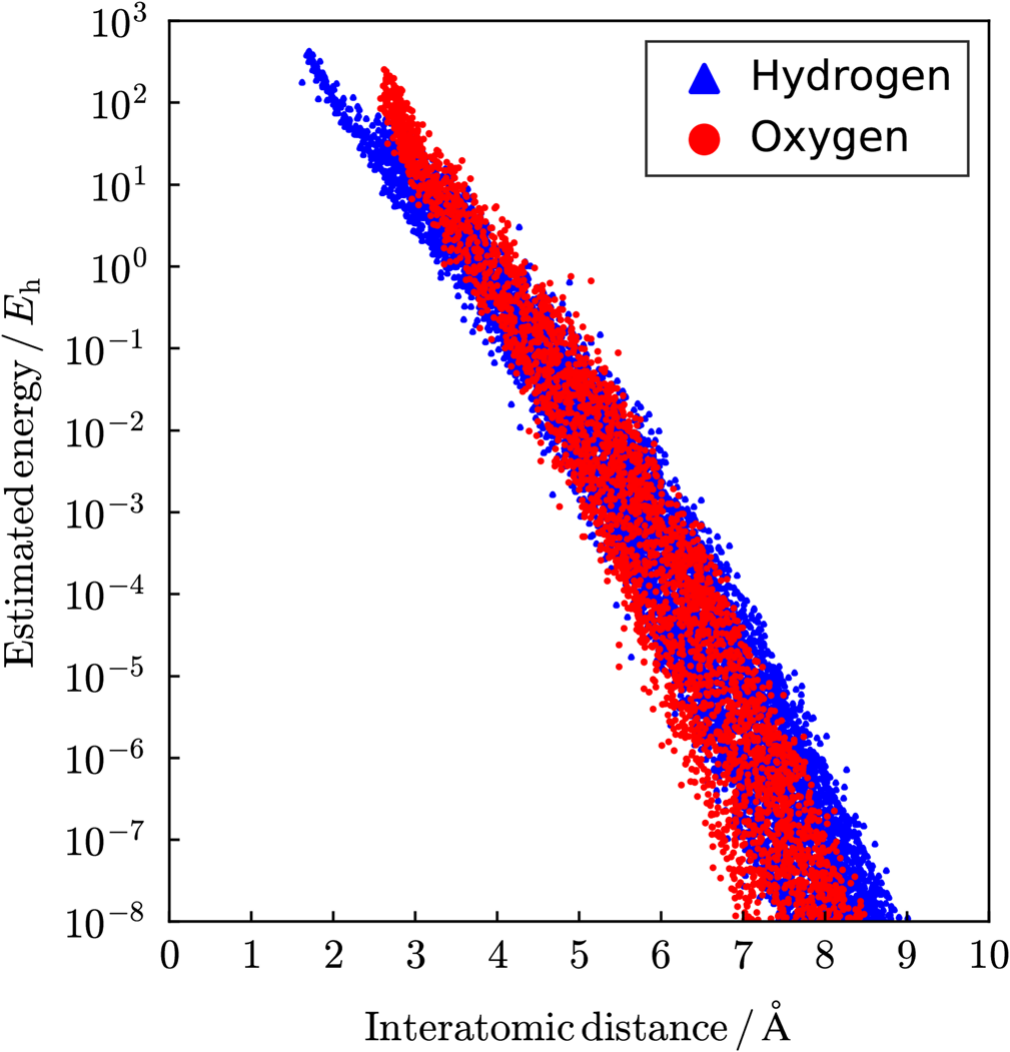
Estimated atomic MP2 energy contributions with respect to the interatomic distance. The blue plots represent the estimated MP2 energy of H atom and the red plots represent of O atom in the buffer region

We first applied the present automated DC‐MP2 method to a cubic system containing 100 water molecules with weight density of 1.0 g cm^−3^. Each water molecule was adopted as a central region in the DC calculation. To assess the performance of the automated DC‐MP2 calculation, the entire system was selected as the initial localization region for every subsystem in the DC‐MP2 calculation. Figure 1 shows the estimated MP2 energy contributions from buffer atom *B* ($e_{B}^{\alpha }$) with respect to its distance from the O atom in the central region. The blue plot represents the value for *B* being an H atom, and the red plot that for *B* being an O atom. The estimated energy contribution decays exponentially as the distance from the central region increases. The slight difference in the slope for H and O atoms in Figure 1 is probably due to the fact that the summation over AOs at the buffer atom in Equation (_9_) runs for the virtual orbital, that is, the charge‐transfer excited configurations from O atoms in donor water to H atoms in acceptor water are more significant than those from acceptor to donor. This behavior was also confirmed for the water dimer system using the intermolecular interaction energy decomposition with the local PNO method.^59^ Note that the estimated energies in Figure 1 for the interatomic distance of 2–3 Å are up to several hundred *E*_h_, which are significantly larger than the total MP2 energy of ∼19 *E*_h_. This is because that the estimated energy ($e_{B}^{\alpha }$) is derived as the upper limit of the atomic MP2 energy contribution. From the following section, the energy threshold in the automated DC‐MP2 method, $e_{\text{thresh}}^{\text{corr}}$, was set to 0.1 μ*E*_h_ unless otherwise noted.

**FIGURE 2:**
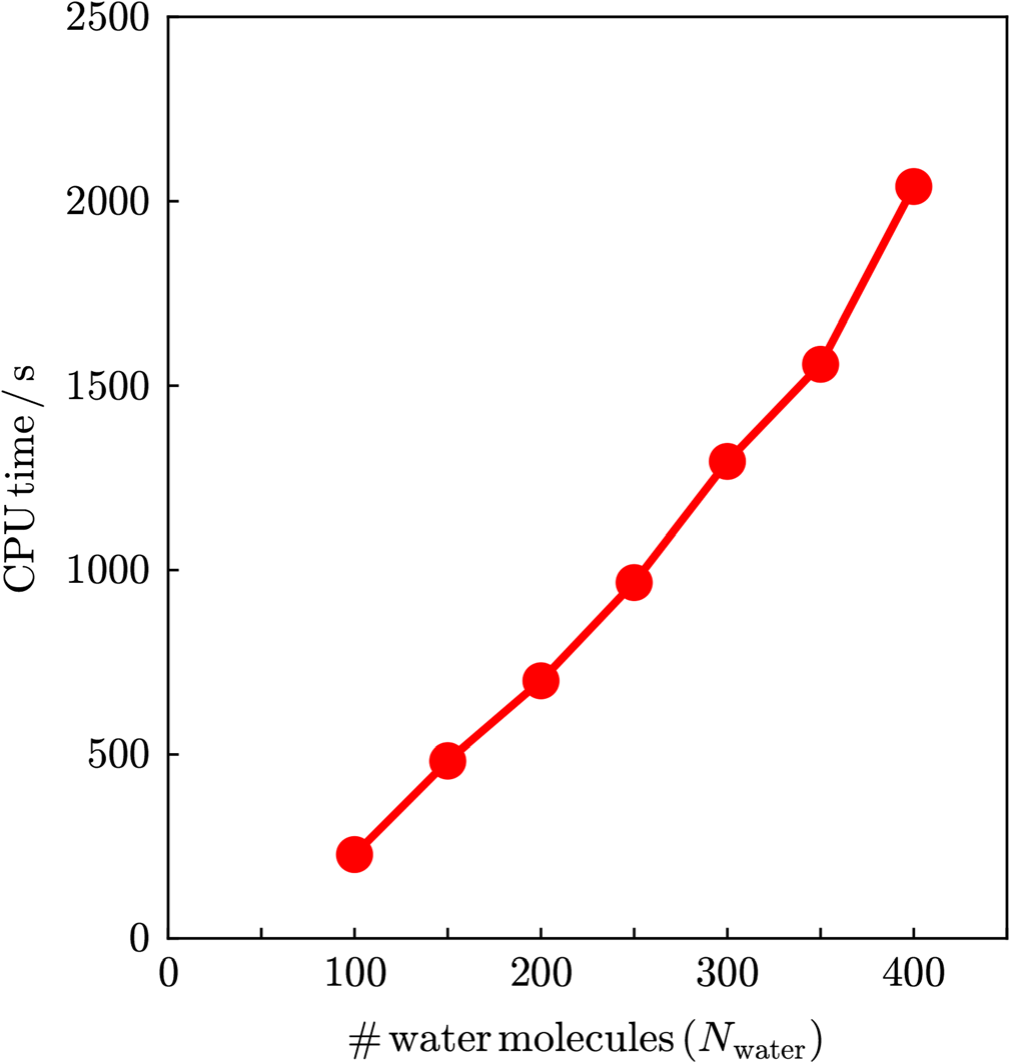
System‐size dependence of the CPU time of the evaluation of $e_{B}^{\alpha }$ for the model system containing *N*_water_ water molecules. The initial buffer size for the DC Hartree–Fock (DC‐HF) calculation was fixed to $r_{b}^{\text{in}}=4.5\text{Å}$ and $r_{b}^{\text{out}}=5.5\text{Å}$

Next, the dependence of the computational time of $e_{B}^{\alpha }$ on the system size was examined, as shown in Figure 2. These were measured using a computer node equipped with two Intel Xeon Gold 5118 CPUs (12 cores, 2.30 GHz) and the average of three measurements was plotted. The initial sizes of the inner and outer buffer regions in the automated DC‐HF calculation were set to $r_{b}^{\text{in}}=4.5\text{Å}$ and $r_{b}^{\text{out}}=5.5\text{Å}$, respectively. The scaling analysis with the double logarithmic plot indicates that the computational time for the evaluation of $e_{B}^{\alpha }$ scales as *O*(*N*_water_^1.5^), which maintains an almost linear‐scaling behavior with respect to the entire system size, as was discussed in Section 2.2.

### 3.3 Accuracy and computational time of the present method

The accuracy and the computational time of the automated DC‐MP2 method were investigated for the cubic water system in Section 3.2. Table 1 shows the energy‐threshold ($e_{\text{thresh}}^{\text{corr}}$) dependence of the DC‐MP2 correlation energy. Following Section 3.2, each water molecule was adopted as a central region and the entire system was selected as the initial localization region. The average and standard deviation (SD) of major axis radii ($〈l_{\text{local}}^{\text{corr}}〉$ and $\sigma \left[l_{\text{local}}^{\text{corr}}\right]$, respectively) are also given in Table 1. For $e_{\text{thresh}}^{\text{corr}}=100\;\mu E_{h}$, the actual correlation energy error per atom is 18.37 μ*E*_h_, which is sufficiently smaller than $e_{\text{thresh}}^{\text{corr}}$. It should be noted that the MP2 energy error decreases systematically as $e_{\text{thresh}}^{\text{corr}}$ decreases, while the dependence is not proportional but rather logarithmic to $e_{\text{thresh}}^{\text{corr}}$. As with the *e*_thresh_ parameter in automated DC‐SCF method,^44^ the smaller $e_{\text{thresh}}^{\text{corr}}$ parameter leads to a larger localization region, which can be confirmed from the average of the major axis radii of all localization regions, $〈l_{\text{local}}^{\text{corr}}〉$. Interestingly, the SD of the major axis radii, $\sigma \left[l_{\text{local}}^{\text{corr}}\right]$, also tends to increase systematically as $e_{\text{thresh}}^{\text{corr}}$ decreases, except for $e_{\text{thresh}}^{\text{corr}}=0.1\;\mu E_{h}$. This fact suggests that the present scheme can effectively aid the selection of the appropriate buffer region for each subsystem in the DC‐MP2 calculation.

**TABLE 1:** $e_{\text{thresh}}^{\text{corr}}$ dependences of the DC‐MP2 correlation energy and the major axis radius for 100 water cluster system

$e_{\text{thresh}}^{\text{corr}}$ (μ*E*_h_)	$E_{\text{corr}}^{\left(2\right)}$ (*E*_h_)	(Diff.) (μ*E*_h_ atom^−1^)	$〈l_{\text{local}}^{\text{corr},\alpha }〉$ (Å)	$\sigma \left[l_{\text{local}}^{\text{corr},\alpha }\right]$ (Å)
100.000	−19.102140	(+18.37)	5.596	0.569
10.000	−19.103891	(+12.54)	6.038	0.589
1.000	−19.104999	(+8.84)	6.380	0.677
0.100	−19.105661	(+6.64)	6.761	0.659
0.010	−19.106160	(+4.97)	7.131	0.681
Standard‐MP2	−19.107652			

Next, we examined the combination of the present automated DC‐MP2 method with the automated DC‐HF calculation. Table 2 shows the dependence of the automated DC‐MP2 energy on the initial DC‐HF inner and outer buffer sizes, $r_{b}^{\text{in}}$ and $r_{b}^{\text{out}}$, the definitions of which are given in our previous paper.^44^ The averages ($〈l_{\text{local}}^{\mathrm{HF}}〉$ and $〈l_{\text{local}}^{\text{corr}}〉$) and the SDs ($\sigma \left[l_{\text{local}}^{\mathrm{HF}}\right]$ and $\sigma \left[l_{\text{local}}^{\text{corr}}\right]$) of the major axis radii among all localization regions in the DC‐HF and DC‐MP2 calculations are also shown. Similar to the results in Ref. 44, the DC‐HF energy error is sufficiently small and almost independent of the initial DC‐HF buffer region. Subsequently, the DC‐MP2 energy error is almost constant (∼8.5 μ*E*_h_ atom^−1^). The average radius of the DC‐HF localization region, $〈l_{\text{local}}^{\mathrm{HF}}〉$, is 7.0–7.2 Å, which is larger than the average radius, 6.761 Å, of the DC‐MP2 localization region for $e_{\text{thresh}}^{\text{corr}}=0.1\;\mu E_{h}$ given in Table 1. A smaller initial DC‐HF buffer size leads to a larger $〈l_{\text{local}}^{\mathrm{HF}}〉$, as was also confirmed in the previous study.^44^ When combined with the automated DC‐HF method, $〈l_{\text{local}}^{\text{corr}}〉$ becomes smaller than its value when the initial localization region is set to be the entire system. Similarly, $\sigma \left[l_{\text{local}}^{\text{corr}}\right]$ is ∼0.14 Å smaller than $\sigma \left[l_{\text{local}}^{\mathrm{HF}}\right]$.

**TABLE 2:** Initial DC‐HF buffer‐size dependence of the automated DC‐MP2 correlation energy and the major axis radius for 100 water cluster system

$r_{b}^{\text{in}}$ (Å)	$r_{b}^{\text{out}}$ (Å)	HF Energy (*E*_h_)	MP2 Energy (*E*_h_)	(Diff.) (μ*E*_h_ atom^−1^)	$〈l_{\text{local}}^{\mathrm{SCF},\alpha }〉$ (Å)	$\sigma \left[l_{\text{local}}^{\mathrm{SCF},\alpha }\right]$ (Å)	$〈l_{\text{local}}^{\text{corr},\alpha }〉$ (Å)	$\sigma \left[l_{\text{local}}^{\text{corr},\alpha }\right]$ (Å)
3.5	4.5	−7601.504443	−19.105142	(+8.37)	7.233	0.840	6.564	0.744
4.0	5.0	−7601.504613	−19.105141	(+8.37)	7.238	0.903	6.538	0.767
4.5	5.5	−7601.504342	−19.105031	(+8.74)	7.161	0.885	6.522	0.743
5.0	6.0	−7601.504417	−19.105000	(+8.84)	7.161	0.905	6.480	0.726
5.5	6.5	−7601.504467	−19.105185	(+8.23)	7.000	0.806	6.427	0.682
Standard		−7601.504673	−19.107652					

*Note*: The energy threshold in the automated DC‐HF calculation is 0.1 μ*E*_h_.

Abbreviation: DC‐HF, DC Hartree–Fock.

Next, we applied the proposed method to a covalently bound system, namely, the chignolin protein with 10 amino acids. The geometry of chignolin was obtained from the protein data bank (PDBID: 1UAO). Hydrogen atoms were added using the Discovery Studio 2017 R2 software.^60^ In the DC calculation, the entire system was divided between the carbonyl C and *α*‐C atoms, and each of the divided systems was treated as a central region. Table 3 shows the $e_{\text{thresh}}^{\text{corr}}$ dependence of the DC‐MP2 energy for chignolin. The entire system was selected as the initial localization region for every subsystem in the DC‐MP2 calculation. For $e_{\text{thresh}}^{\text{corr}}=100\;\mu E_{h}$, the actual correlation energy error per atom is 2.82 μ*E*_h_, which is sufficiently smaller than $e_{\text{thresh}}^{\text{corr}}$. As was also confirmed in the case of the water system, the MP2 energy error decreases systematically as $e_{\text{thresh}}^{\text{corr}}$ decreases. Again, the dependence of the error on $e_{\text{thresh}}^{\text{corr}}$ is rather logarithmic. The smaller $e_{\text{thresh}}^{\text{corr}}$ leads to the larger $〈l_{\text{local}}^{\text{corr}}〉$, while it leads to the smaller $\sigma \left[l_{\text{local}}^{\text{corr}}\right]$, contrary to the case of water system. Comparing Table 3 with Table 1, $〈l_{\text{local}}^{\text{corr}}〉$ of chignolin is about 1.0 Å larger than that of the water system for the same $e_{\text{thresh}}^{\text{corr}}$ parameter, reflecting the delocalized electronic nature in the covalently bound system.

**TABLE 3:** $e_{\text{thresh}}^{\text{corr}}$ dependences of the DC‐MP2 correlation energy and the major axis radius for chignolin

$e_{\text{thresh}}^{\text{corr}}$ (μ*E*_h_)	$E_{\text{corr}}^{\left(2\right)}$ (*E*_h_)	(Diff.) (μ*E*_h_ atom^−1^)	$〈l_{\text{local}}^{\text{corr},\alpha }〉$ (Å)	$\sigma \left[l_{\text{local}}^{\text{corr},\alpha }\right]$ (Å)
100.000	−11.194529	(+2.82)	7.003	0.671
10.000	−11.194689	(+1.67)	7.185	0.598
1.000	−11.194770	(+1.08)	7.530	0.614
0.100	−11.194828	(+0.66)	7.629	0.597
0.010	−11.194847	(+0.52)	7.726	0.564
Standard‐MP2	−11.194919			

Next, we combined this with the automated DC‐HF calculation. Table 4 shows the dependence of the DC‐MP2 energy on the initial DC‐HF buffer size. The automated DC‐HF energy error for chignolin is smaller than that for the water system and almost independent of the initial DC‐HF buffer region, while the radius of the DC‐HF localization region (∼7.5 Å) is about 1 Å greater than for the water system (∼6.5 Å). Subsequently, the DC‐MP2 energy error is also almost constant (∼0.7 μ*E*_h_ atom^−1^). For this small protein system, in contrast to the result in Table 2 for the water system, the SD of the sizes of the localization regions for the MP2 calculation is larger than that for the HF calculation. This is because the entire size of the chignolin system is so small that the localization region for every subsystem is close to the entire system. The present method was also tested on the *β*‐strand glycine oligomer (GLY)_20_, and the result of the calculation are given in Table 5. In Table 5, the DC‐MP2 calculations with different $e_{\text{thresh}}^{\text{corr}}$ were performed to confirm that the present automated DC‐MP2 energy error depends primarily on $e_{\text{thresh}}^{\text{corr}}$ and hardly on the initial buffer radii. For this stretched system, the SD of the localization region sizes for the MP2 calculation is smaller than that for the HF calculation, while the energy error is similar to the result in Table 4. As well as the case of water system, the smaller $e_{\text{thresh}}^{\text{corr}}$ leads to the larger $〈l_{\text{local}}^{\text{corr}}〉$ and $\sigma \left[l_{\text{local}}^{\text{corr}}\right]$.

**TABLE 4:** Initial DC‐HF buffer‐size dependence of the automated DC‐MP2 correlation energy and the major axis radius for chignolin

$r_{b}^{\text{in}}$ (Å)	$r_{b}^{\text{out}}$ (Å)	HF Energy (*E*_h_)	MP2 Energy (*E*_h_)	(Diff.) (μ*E*_h_ atom^−1^)	$〈l_{\text{local}}^{\mathrm{SCF},\alpha }〉$ (Å)	$\sigma \left[l_{\text{local}}^{\mathrm{SCF},\alpha }\right]$ (Å)	$〈l_{\text{local}}^{\text{corr},\alpha }〉$ (Å)	$\sigma \left[l_{\text{local}}^{\text{corr},\alpha }\right]$ (Å)
3.5	4.5	−3799.529116	−11.194860	(+0.43)	8.248	0.550	7.606	0.620
4.0	5.0	−3799.528978	−11.194810	(+0.79)	8.121	0.626	7.606	0.620
4.5	5.5	−3799.528977	−11.194825	(+0.68)	8.174	0.562	7.582	0.598
5.0	6.0	−3799.528978	−11.194820	(+0.71)	8.304	0.625	7.606	0.620
5.5	6.5	−3799.528978	−11.194814	(+0.76)	8.151	0.508	7.606	0.620
Standard		−3799.528980	−11.194919					

*Note*: The energy threshold in the automated DC‐HF calculation is 0.1 μ*E*_h_.

Abbreviation: DC‐HF, DC Hartree–Fock.

**TABLE 5:** Initial DC‐HF buffer‐size dependence of the automated DC‐MP2 energy and the major axis radius for the *β*‐strand glycine oligomer (GLY)_20_

$r_{b}^{\text{in}}$ (Å)	$r_{b}^{\text{out}}$ (Å)	HF Energy (*E*_h_)	MP2 Energy (*E*_h_)	(Diff.) (μ*E*_h_ atom^−1^)	$〈l_{\text{local}}^{\mathrm{SCF},\alpha }〉$ (Å)	$\sigma \left[l_{\text{local}}^{\mathrm{SCF},\alpha }\right]$ (Å)	$〈l_{\text{local}}^{\text{corr},\alpha }〉$ (Å)	$\sigma \left[l_{\text{local}}^{\text{corr},\alpha }\right]$ (Å)
$e_{\text{thresh}}^{\text{corr}}$ = 100 μ*E*_h_								
3.5	4.5	−4211.847790	−11.932344	(+1.01)	10.469	1.534	7.488	0.910
4.0	5.0	−4211.847790	−11.932345	(+1.00)	10.501	1.450	7.488	0.910
4.5	5.5	−4211.847790	−11.932345	(+1.01)	10.469	1.534	7.488	0.910
5.0	6.0	−4211.847790	−11.932345	(+1.01)	10.469	1.534	7.488	0.910
5.5	6.5	−4211.847790	−11.932345	(+1.01)	10.469	1.534	7.488	0.910
$e_{\text{thresh}}^{\text{corr}}$ = 0.1 μ*E*_h_								
3.5	4.5	−4211.847790	−11.932431	(+0.40)	10.469	1.534	8.603	1.136
4.0	5.0	−4211.847790	−11.932433	(+0.39)	10.501	1.450	8.603	1.136
4.5	5.5	−4211.847790	−11.932432	(+0.40)	10.469	1.534	8.603	1.136
5.0	6.0	−4211.847790	−11.932432	(+0.39)	10.469	1.534	8.603	1.136
5.5	6.5	−4211.847790	−11.932432	(+0.40)	10.469	1.534	8.603	1.136
Standard		−4211.847819	−11.932489					

*Note*: The energy threshold in the automated DC‐HF calculation is set to 0.1 μ*E*_h_ and that in the automated DC‐MP2 calculation is set to 100 or 0.1 μ*E*_h_.

Abbreviation: DC‐HF, DC Hartree–Fock.

**FIGURE 3:**
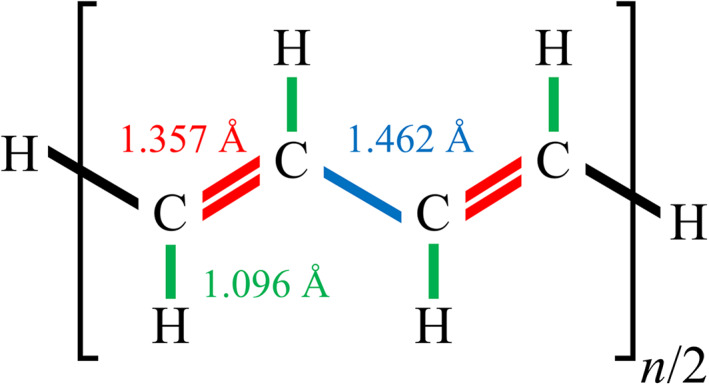
Structure of polyacetylene chain system, C_2*n*_H_2*n*+2_

Finally, the present method was applied to the conjugated polyacetylene chain C_2*n*_H_2*n* + 2_, shown in Figure 3. All atoms were placed in a plane and the C—C, C=C, and C—H bond lengths were fixed at 1.462, 1.357, and 1.096 Å, respectively. Each C_2_H_2_ (or C_2_H_3_ for edges) unit divided at the C—C single bond was treated as a central region. Table 6 shows the system‐size dependence of the standard and DC‐MP2 energies. For the automated DC calculations, the initial sizes of the inner and outer buffer regions in the automated DC‐HF calculation were set to $r_{b}^{\text{in}}=5.0\text{Å}$ and $r_{b}^{\text{out}}=6.5\text{Å}$, respectively. To avoid division of the localization region at C=C double bond, each C_2_H_2_ (or C_2_H_3_) unit was treated as one piece, that is, a unit was extracted from the DC‐MP2 localization region only when all the estimated MP2 correlation energies, $\left\{e_{B}^{\alpha }\right\}$, for the atoms in the unit were smaller than the threshold, $e_{\text{thresh}}^{\text{corr}}$ (analogous to the BUFTYP = RADSUB option of $DANDC input group in GAMESS program). The DC‐MP2 energy error per atom is almost constant for *n* ≥ 30. It was demonstrated that the correlation energy error can be controlled with the present method, even for conjugated systems.

**TABLE 6:** The system‐size dependence of the MP2 electron correlation energy in the standard MP2 and automated DC‐MP2 calculations for polyacetylene chain system, C_2*n*_H_2*n*+2_

# of C atoms	Standard‐MP2	Auto. DC‐MP2	
	Energy (*E*_h_)	Energy (*E*_h_)	(Diff.) (μ*E*_h_ atom^−1^)
10	−1.266346	−1.266346	(+0.00)
20	−2.533020	−2.532799	(+5.25)
30	−3.799773	−3.799303	(+7.58)
40	−5.066529	−5.065806	(+8.81)
50	−6.333285	−6.332309	(+9.56)
60	−7.600041	−7.598813	(+10.06)
70	−8.866797	−8.865319	(+10.40)
80	−10.133553	−10.131822	(+10.68)
90	−11.400309	−11.398327	(+10.89)
100	−12.667065	−12.664831	(+11.06)

**FIGURE 4:**
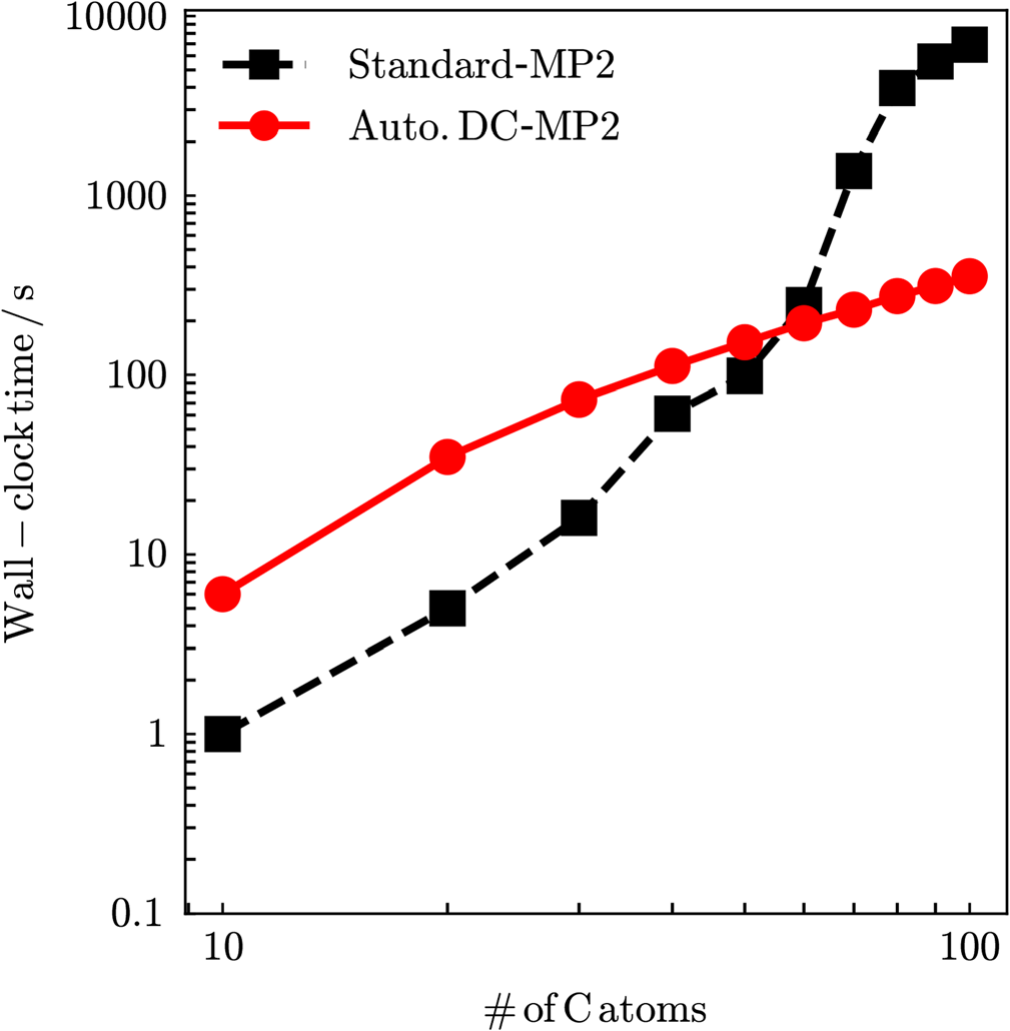
System‐size dependence of the Wall‐clock time of the standard MP2 and the automated DC‐MP2 calculations for polyacetylene chain system containing *n* carbon atoms C_2*n*_H_2*n*+2_. Black dashed line: standard‐MP2; solid red line: automated DC‐MP2

For this conjugated system, the dependence of the computational time on the system size was also examined, as shown in Figure 4. The computational time for the MP2 calculation was measured using a computer node equipped with two Intel Xeon E5–2667 CPUs (8 cores, 3.20 GHz), and the average of three measurements was plotted. For comparison, the time required for the standard MP2 calculation was also plotted. The CODE = IMS program^61^ specified in the $MP2 input group implemented in the GAMESS package was used. The automated DC‐MP2 calculation shows a faster computational time than that of the standard MP2 calculation for *n* ≥ 30. The scaling analysis with the double logarithmic plot for *n* ≥ 40 indicates that the computational time for the standard MP2 scales as *O*(*n* ^2.5^), while that for the present automated DC‐MP2 method scales as *O*(*n* ^1.1^). It is confirmed that the linear‐scaling behavior of the DC‐MP2 method is preserved even with the present automation scheme.

**FIGURE 5:**
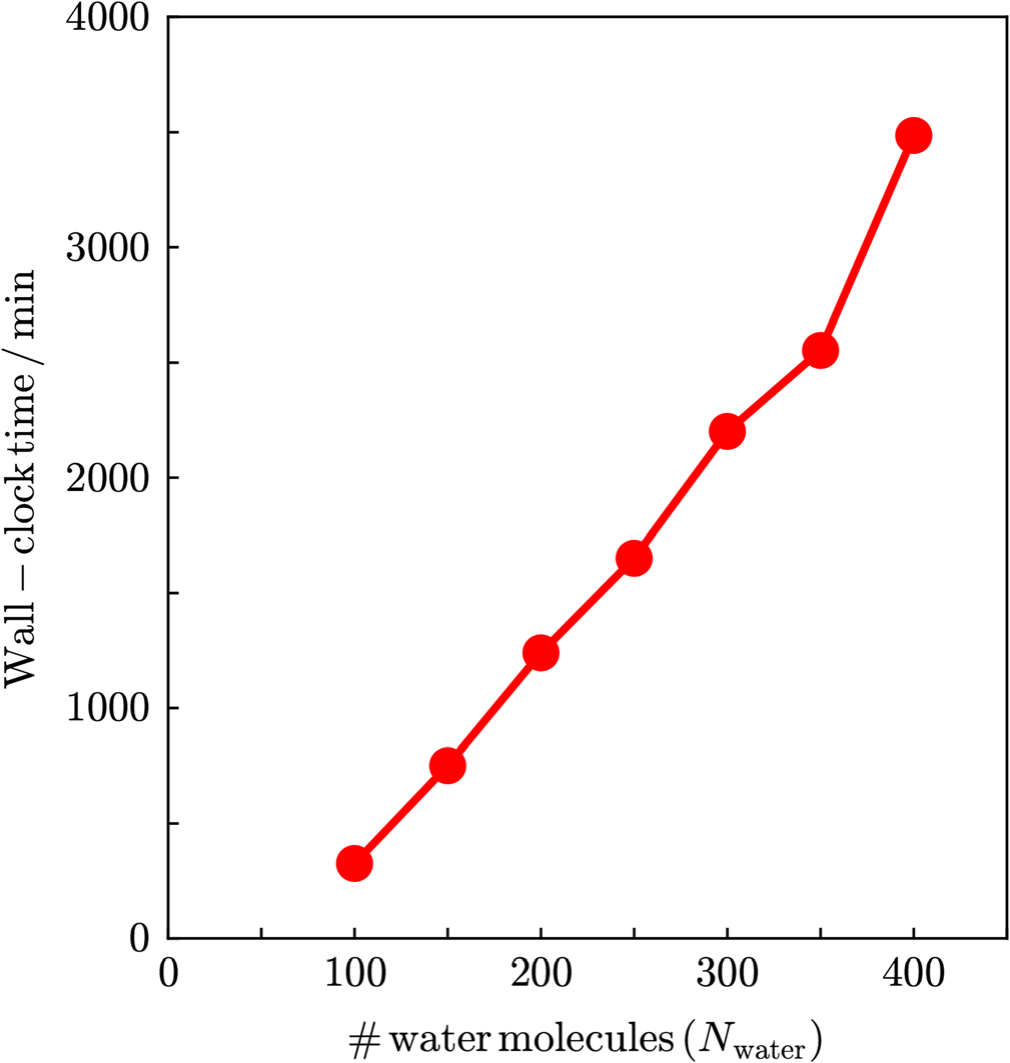
System‐size dependence of the Wall‐clock time of the automated DC‐MP2 calculations for the model system containing *N*_water_ water molecules. The initial sizes of the inner and outer buffer regions in the automated DC Hartree–Fock (DC‐HF) calculation were set to $r_{b}^{\text{in}}=4.5\text{Å}$ and $r_{b}^{\text{out}}=5.5\text{Å}$, respectively. The energy threshold in the automated DC‐MP2 method, $e_{\text{thresh}}^{\text{corr}}$, was set to 10 μ*E*_h_

The scaling analysis was also conducted for three‐dimensional water cluster systems. Figure 5 shows the dependence of the wall‐clock computational time for the DC‐MP2 calculation on the number of water molecules, *N*_water_. The times were measured using a computer node equipped with two Intel Xeon Gold 5118 CPUs (12 cores, 2.30 GHz), and the average of three measurements was plotted. The initial sizes of the inner and outer buffer regions in the automated DC‐HF calculation were set to $r_{b}^{\text{in}}=4.5\text{Å}$ and $r_{b}^{\text{out}}=5.5\text{Å}$, respectively. The energy threshold in the automated DC‐MP2 method, $e_{\text{thresh}}^{\text{corr}}$, was set to 10 μ*E*_h_. The scaling analysis with the double logarithmic plot indicates that the computational time for the present automated DC‐MP2 method scales as *O*(*N*_water_^1.6^), which indicates that the present method also achieves near‐linear scaling computational time even for three‐dimensional systems.

## 4 CONCLUDING REMARKS

In this study, we have proposed an automatic determination scheme for the buffer region in the DC‐MP2 calculation. This method is based on a subsystem MP2 correlation energy contribution from each atom in the buffer region, which is estimated with the help of the AO‐Laplace MP2 method and the Schwarz inequality. Because the appropriate size of the buffer region in the DC‐MP2 calculation can be smaller than that in the DC‐HF calculation, as suggested in a previous paper,^36^ the present scheme reduces the buffer region from the prior DC‐HF calculation. We applied the present method to a 100 water cluster system and the chignolin system, and confirmed that the estimated DC‐MP2 energy error can be systematically reduced as the energy threshold, $e_{\text{thresh}}^{\text{corr}}$, decreases. We also confirmed that the linear‐scaling behavior of the DC‐MP2 method is preserved even with the present automation scheme.

Since the MP2 amplitude is known to provide a good guess for the CC method in many cases, the proposed automation scheme is straightforwardly applicable to the DC‐CC method.^37^, ^38^, ^39^ Improvements in the accuracy of the correlation energy contributions from buffer atoms are also desirable, especially for delocalized systems. The use of the inequality test proposed by Thompson et al.^62^ instead of the Schwarz inequality would be one way to provide this improvement.

## Figures and Tables

**FIGURE A1:**
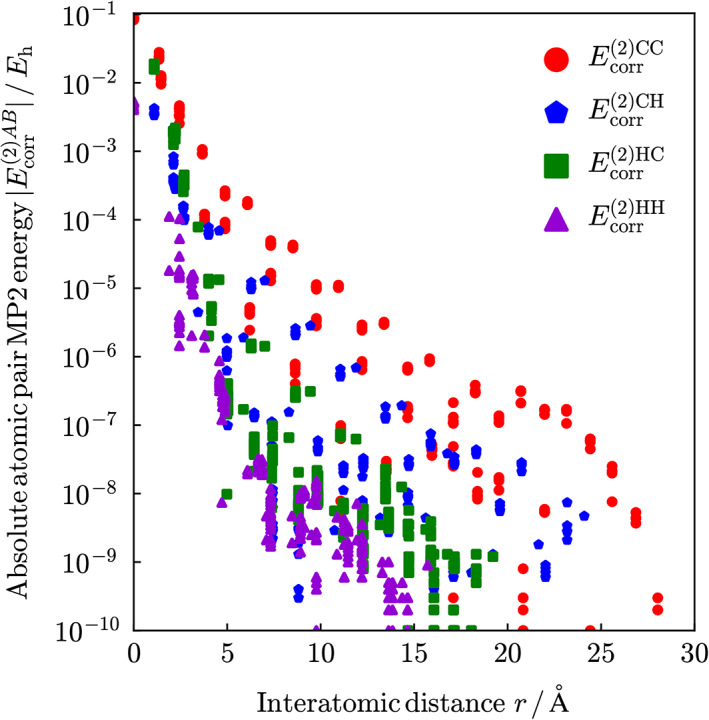
The absolute atomic pair MP2 correlation energy contribution with respect to the interatomic distance. The circle, pentagon, square and triangle plots represent the MP2 correlation energy contribution for C—C, C—H, H—C and H—H atomic pairs, respectively

## Data Availability

The data that support the findings of this study are available from the corresponding author upon reasonable request.

## MP2 CORRELATION ENERGY DENSITY FOR BONDS

Here, we propose a scheme to partition the standard MP2 energy into atom‐pair (bond) contributions to demonstrate the local character of the MP2 correlation. The scheme is related to the bond EDA proposed by Nakai and coworkers.^63^

The MP2 correlation energy can be divided into contributions from the atomic pair, expressed as

(A1) $$\Delta E_{\text{corr}}^{\left(2\right)}=\sum_{A,B}\Delta E_{\text{corr}}^{\left(2\right)\mathrm{AB}}$$

(A2) $$\Delta E_{\text{corr}}^{\left(2\right)\mathrm{AB}}=\sum_{i,j}^{\mathrm{occ}}\sum_{a,b}^{\mathrm{vir}}\sum_{\mu \;\in \;A}\sum_{\nu \;\in \;B}\frac{C_{\mathrm{\mu i}}C_{\mathrm{\nu a}}\left(\mu \nu |\mathrm{jb}\right)}{\varepsilon_{i}+\varepsilon_{j}-\varepsilon_{a}-\varepsilon_{b}}\left[2\left(\mathrm{ai}|\mathrm{bj}\right)-\left(\mathrm{aj}|\mathrm{bi}\right)\right]$$

Here, we have adopted the overlap separation instead of the electron‐pair separation^52^, ^53^, ^54^ to exploit the local nature of the MP2 correlation. This form is also consistent with $\Delta E_{B}^{\alpha \left(2\right)}$, Equation (_9_). Note that $\Delta E_{\text{corr}}^{\left(2\right)\mathrm{AB}}$ is different from $\Delta E_{\text{corr}}^{\left(2\right)\mathrm{BA}}$ because atoms *A* and *B* in $\Delta E_{\text{corr}}^{\left(2\right)\mathrm{AB}}$ are associated with the occupied and virtual orbitals, respectively.

The atom‐pair MP2 correlation energies, $\Delta E_{\text{corr}}^{\left(2\right)\mathrm{AB}}$, were evaluated for C_30_H_32_ polyene system with 6‐31G(d) basis set. Figure A1 shows the dependence of $\Delta E_{\text{corr}}^{\left(2\right)\mathrm{AB}}$ on the distance between the *A* and *B* atoms, *r*. Different color plots indicate different combinations of elements for atoms *A* and *B*. Overall, the atom‐pair contribution decreases exponentially with respect to the distance, although that for $\Delta E_{\text{corr}}^{\left(2\right)\mathrm{CC}}$ has small hump around *r* = 20 Å. Reflecting the small number of correlated electrons around H atom, $\Delta E_{\text{corr}}^{\left(2\right)\mathrm{HH}}$ has the smallest contribution at the same distance *r*. $\Delta E_{\text{corr}}^{\left(2\right)\mathrm{CH}}$ is larger than $\Delta E_{\text{corr}}^{\left(2\right)\mathrm{HC}}$, probably due to more significant contribution of the charge‐transfer excitation configurations from the electron‐rich C atoms to the electron‐deficient H atoms, similar to the discussion on the water system (see Section 3.2).
